# Differential Cellular Dynamics of Platelet‐Derived Growth Factor Receptor β Cells and Macrophages After Complete Spinal Cord Transection in Neonatal and Juvenile Rats

**DOI:** 10.1111/ejn.70745

**Published:** 2026-10-29

**Authors:** Masahito Takiguchi, Reina Tamaji, Kengo Funakoshi

**Affiliations:** ^1^ Department of Neuroanatomy Yokohama City University School of Medicine Yokohama Japan

**Keywords:** collagen, fibroblasts, juvenile, macrophage, neonate, spinal cord injury

## Abstract

Lesion morphology after complete spinal cord transection (CST) varies depending on the time of injury. Following CST, the stumps are connected by fibrous tissue in adult and juvenile rats, whereas they are connected by thin meningeal tissue in neonatal rats. Here, we compared the cellular dynamics after CST in neonatal and juvenile rats to investigate factors contributing to the morphologic differences between the two groups. The intensity of PDGFRβ‐positive structures associated with fibrotic scar formation at the lesion core was significantly higher in juvenile CST rats than in neonatal CST rats. In neonatal rats, the intensity gradually decreased, whereas in juvenile rats it peaked at 7 days and then decreased. Pro‐inflammatory CD11c‐positive macrophages were abundant in the lesion core 2 days after CST, then rapidly decreased in both neonatal and juvenile CST rats. Anti‐inflammatory CD163‐positive macrophages in the lesion core were significantly more abundant in neonatal CST rats than in juvenile CST rats and remained elevated until 14 days after CST. Neonatal CST rats exhibited less fibroblast accumulation and higher CD163‐positive macrophage density in the lesion core compared with juvenile CST rats. These differences may be associated with morphologic differences in the lesion core between the two groups. CD163‐positive macrophages in the neonatal CST rats may primarily promote meningeal remodeling rather than fibrous tissue proliferation leading to fibrous scar formation.

## Graphical abstract

Following CST, the stumps are connected by fibrous tissue in adult and juvenile rats, whereas they are connected by thin meningeal tissue in neonatal rats. The intensity of PDGFRβ‐positive fibroblasts associated with fibrotic scar formation at the lesion site was significantly higher in juvenile CST rats than in neonatal CST rats. Antti‐inflammatory M2c macrophages (CD163‐positive) at the lesion site were significantly more abundant in neonatal CST rats than in juvenile CST rats. These differences may be associated with morphologic differences at the lesion site between the two groups. M2c macrophages in the neonatal CST rats may primarily promote meningeal remodeling rather than fibrous tissue proliferation leading to fibrous scar formation.

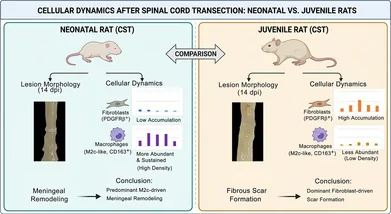

**Abbreviations**
ANOVA: Analysis of variance
CST: Complete spinal cord transection
DAPI: 4′6‐Diamino‐2‐phenylinodole
dpi: Days post injury
ECM: Extracellular matrix
GFAP: Glial fibrillary acidic protein
HEXB: Hexosaminidase subunit beta
IL: Interleukin
MMP: Matrix metalloproteinase
PB: Phosphate buffer
PBS: Phosphate‐buffered saline
PBST: PBS with Tween 20
PDGFRβ: Platelet‐derived growth factor receptor β
PFA: Paraformaldehyde
SCI: Spinal cord injury
TGF: Transforming growth factor

## 1 Introduction

Complete spinal cord transection (CST) at the thoracic level in adult rats causes permanent loss of hindlimb locomotor function in adult and juvenile rats, whereas neonatal rats exhibit spontaneous recovery of hindlimb motor function (Stelzner et al. 1975; Yuan et al. 2013). The critical period for this functional recovery is between 10 and 15 days of age (Weber and Stelzner 1977; Yuan et al. 2013). The mechanisms underlying spontaneous recovery in neonatal rats remain unclear. Previous studies demonstrated enhanced primary afferent projections to the lumbar spinal motor area, including the ventral horn and intermediate zone, following neonatal CST at the thoracic level (Takiguchi et al. 2015, 2017, 2025, 2026). In addition to increased primary afferent projections, high sustained synaptic plasticity in spinal neurons may contribute to motor function recovery in neonatal rats (Takiguchi et al. 2022, 72,025, 2026). Through these compensatory mechanisms, neonatal rats that undergo CST at postnatal day 5 (P5) recover motor function to a BBB score of 8.5 after 4 weeks (Takiguchi et al. 2015). Due to the large cavity formed after CST, however, axonal regeneration across the injury site does not occur in neonatal rats, which may limit further recovery of motor function Tillakaratne et al. 2010; Yuan et al. 2013). Furthermore, no distinct tissue repair occurs at the injury site after neonatal CST; rather than forming a fibrotic scar, the entire lesion core narrows, leaving the proximal and distal stumps connected by a thin membranous tissue (Yuan et al. 2013).

These features of CST in neonatal rats sharply contrast with those observed after spinal cord contusion injury. In mice, fibronectin secreted by microglia accumulating at the contusion site forms a bridge between the proximal and distal stumps, along which long descending axons can extend. This scar‐free tissue repair contributes to robust recovery of motor function (Li et al. 2020). The response to CST in neonatal rats also differs markedly from that observed after CST in adult rats. In adult rats, fibrous scar tissue and blood‐filled cavities form at the injury site within 2 weeks after CST, effectively bridging the proximal and distal stumps (Wiksten et al. 2004).

The mechanisms underlying the significant morphologic differences of the lesion core between neonatal CST and adult CST are essentially unknown. Whereas adult CST induces fibroblast migration and proliferation at the injury site, leading to fibrotic scar formation, neonatal CST appears to promote tissue remodeling without fibroblast migration and proliferation. In this study, we quantitatively investigated the spatiotemporal changes in fibroblast dynamics following neonatal CST and compared them with those after juvenile CST, which induces fibrotic scar formation similar to that observed after adult CST. Platelet‐derived growth factor receptor β (PDGFRβ) was used as a marker for fibroblasts and other mesenchyme‐derived cells including pericytes and vascular mesenchymal cells, all of which contribute to fibrotic scar formation.

Macrophages and microglia are involved in fibroblast dynamics after spinal cord injury (SCI) in mice, including migration to the injury site, extracellular matrix (ECM) formation, and fibrotic scar formation (Zhu et al. 2015; Funk et al. 2016; Shan et al. 2024). Macrophages are broadly classified into pro‐inflammatory M1 macrophages or anti‐inflammatory M2 macrophages, with M2 macrophages further subdivided into M2a, M2b, M2c, and M2d. During wound healing in somatic organs, M2a macrophages are primarily involved in cell recruitment and proliferation, whereas M2c macrophages are involved in tissue repair and remodeling (Gensel and Zhang 2015). Previous studies suggested that both M1 and M2 macrophages are activated in the early phase after adult SCI (Donnelly and Popovich 2008), and that M2 macrophages, particularly M2a, play a major role in promoting fibroblast migration and proliferation into the lesion core (Gensel and Zhang 2015; Li et al. 2021). M2c participates in tissue repair and somatic organ remodeling, but their role following SCI is unclear. It is considered that M2c macrophages are not activated after SCI because normal remodeling does not occur (Gensel and Zhang 2015). After neonatal CST, on the other hand, tissue repair is promoted without fibroblast proliferation, suggesting that M2c macrophages play a crucial role. Here, we investigated and compared the dynamics of M1 and M2c macrophages following CST in neonatal and juvenile rats to explore their potential contribution to differences in lesion morphology. Although microglia were not distinguished from macrophages in this analysis as, their presence at the injury site was examined separately using microglia‐specific markers.

## 2 Materials and Methods

### 2.1 Materials

Wistar neonatal and juvenile rats (*n* = 20, Japan SLC, Hamamatsu, Japan) and their dams were used in the present study. The sample size was determined based on statistically valid methods and the principle of reduction in animal welfare. All experimental procedures were performed according to the standards established by the NIH Health Guide for the Care and Use of Laboratory Animals and the Policies on the Use of Animals and Humans in Research. The protocols were approved by the Institutional Animal Care and Use Committee of the Animal Research Center, Yokohama City University Graduate School of Medicine (approval no. F‐A‐24‐047).

### 2.2 Complete Spinal Cord Transection

Spinal cords of neonatal rats (P5, *n* = 25) or juvenile rats (P20: *n* = 25) were transected at the 10th thoracic level (T10) as previously described (Takiguchi et al. 2015). Briefly, the skin, fascia, and muscles were incised, exposing the vertebral discs of the 9th to 10th thoracic vertebrae. Under isoflurane gas (1.5%–2.0%) anesthesia, spinal cords were completely transected with small spring microscissors following partial laminectomy (T9–T10). After confirming the completeness of the spinal transection, bleeding was stopped with Spongel (LTL Pharma Co. Ltd., Tokyo, Japan). The muscles, fascia, and skin were closed in layers with 5‐0 nylon sutures. After surgery, all rats were gently cleaned with gauze soaked in 70% ethanol to remove blood odor and returned to their home cages after recovery from anesthesia. All rats with CST were housed individually in polycarbonate cages in a room maintained at 25 ± 1 °C, with a 07:00 on/19:00 off light cycle.

### 2.3 Fixation and tissue preparation

Rats that underwent CST at P5 (P5ST group) or P20 (P20ST groups) were deeply anaesthetized with isoflurane at 2 days post injury (dpi: *n* = 5), 4 dpi (*n* = 5), 7 dpi (*n* = 5), 10 dpi (*n* = 5), or 14 dpi (*n* = 5), and transcardially perfused with normal saline followed by 4% paraformaldehyde (PFA) in 0.1 M phosphate buffer (PB). The spinal cords were dissected and postfixed with 4% PFA overnight at 4 °C. The tissues were cryoprotected in 25% sucrose for 2 days and embedded in OCT compound via 2‐methylbutane (isopentane) in liquid N_2_. Sections of the spinal cord containing the lesion core and the surrounding area from the P5ST (*n* = 25) and P20ST (*n* = 25) groups were serially sectioned at 20 μm using a cryostat (CM3050 S, Leica, Nussloch, Germany). Serial sections were collected as a 1‐in‐5 series, with every fifth section included for analysis.

### 2.4 Immunohistochemistry

A series of sections was incubated in a moist chamber overnight at 4 °C with a mixture of (1) rabbit polyclonal IgG antibody against PDGFRβ (1:200, ab231480, abcam, Cambridge, United Kingdom, RRID: not registered), and goat polyclonal IgG antibody against collagen I (1:50, AB758, MilliporeSigma, Billerica, Massachusetts, United States, RRID:AB_92249): (2) rabbit polyclonal IgG antibody against CD11c (1:100, ab175888, abcam, RRID: not registered), and goat polyclonal IgG antibody against glial fibrillary acidic protein (GFAP: 1:500, ab53554, abcam, RRID:AB_880202): (3) rabbit polyclonal IgG antibody against CD163 (1:100, ab227781, abcam, RRID: not registered), and goat polyclonal IgG antibody against GFAP (1:500, abcam), diluted with 1% normal donkey serum, 0.2% bovine serum albumin, and 0.1% NaN3 in 0.025 M phosphate‐buffered saline (PBS) with Tween 20 (PBST). Some sections from the P5ST group at 2, 4, and 7 dpi were incubated overnight with rabbit polyclonal IgG antibody against Sall1 (1:150, 27,761–1‐AP, Proteintech, Rosemont, Illinois, United States) or hexosaminidase subunit beta (HEXB, 1:150, 16229‐1‐AP, Proteintech). Both Sall1 and HEXB are specific markers of microglia (Buttgereit et al. 2016; Masuda et al. 2020). The sections were rinsed several times with 0.025 M PBST and then incubated for 2 h at room temperature with a mixture of secondary antibodies: Alexa Fluor 488‐conjugated donkey anti‐rabbit IgG (10 μg/mL; Jackson ImmunoResearch Laboratories, West Grove, Pennsylvania, United States) and cyanine Cy3‐conjugated donkey anti‐goat IgG (10 μg/mL; Jackson ImmunoResearch Laboratories) diluted with 1% normal donkey serum, 0.2% bovine serum albumin, and 0.1% NaN3 in 0.025 M PBST. The antibody specificity was verified by incubation with 0.5% normal mouse serum or 0.5% normal rat serum (both from Jackson ImmunoResearch Laboratories) instead of the primary antibodies. The slides were rinsed 5 times with PBS, counterstained with 4′6‐diamino‐2‐phenylinodole (DAPI) to visualize cell nuclei, and cover‐slipped with slow‐fade reagent (SlowFade Diamond Antifade Mountant, S36936, Life Technologies Corporation, Carlsbad, California, United States).

### 2.5 Image Acquisition and Analysis

All spinal sections were digitally photographed using a Keyence BIOREVO microscope (BZ‐9000, Keyence, Osaka, Japan) and transferred to Adobe Photoshop CS (Adobe, San Jose, California, United States). The triple‐labeled images were converted to pseudocolor to facilitate visualization, and contrast and brightness were adjusted using Adobe Photoshop.

### 2.6 Quantitative Analysis of PDGFRβ‐Positive Structures and Macrophages in the Lesion Core

The lesion core was defined as areas containing PDGFRβ‐positive structures and collagen I, or areas negative for GFAP immunoreactivity. The morphology of PDGFRβ‐positive structures was highly variable, making it difficult to count the number of cells. The immunofluorescence intensity of PDGFRβ‐positive structures was quantified in each section with ImageJ software, and the fluorescence intensity normalized to the lesion area (volume/mm^2^) was calculated. The area‐normalized immunofluorescence intensity of PDGFRβ at each dpi stage in each group was averaged, and the temporal changes in PDGFRβ‐positive structures in the lesion area were quantitatively analyzed. Mean fluorescence intensity is expressed as mean ± SEM.

The number of macrophages in the lesion core was counted in each section using a blinded method, and cell density (cell number/mm^2^) was calculated by dividing the cell count by the lesion area. The average cell density at each dpi stage in each group was calculated, and the temporal changes in the cell density were quantitatively analyzed. Average density is expressed as mean ± SEM.

### 2.7 Statistical Analysis

Statistical analysis of PDGFRβ immunofluorescence intensity and macrophage cell densities was performed using two‐way analysis of variance (ANOVA) to access the differences between the CST groups and time after CST, using SPSS software (IBM, Armonk, New York, United States), which applies a sum‐to‐zero constraint for factor‐level comparisons. *p* Values of less than 0.05 were considered significant. Post hoc comparison were performed using Tukey's honestly significant difference (HSD) test. Two‐tailed *p* values of less than 0.05 were considered significant. Additional analyses were performed with one‐way ANOVA and the post hoc Game‐Howell test using SPSS software (IBM).

## 3 Results

### 3.1 Gross and Microscopic Morphology of the Lesion Core After CST

In the P5ST group, the lesion core gradually thinned from 4 dpi, with the rostral and caudal ends connected only by membranous structures (Figure 1A–D). In the P20ST group, the lesion core did not thin from 4 dpi, and the rostral and caudal ends were connected by distinct scar tissue (Figure 1E–H).

**FIGURE 1:**
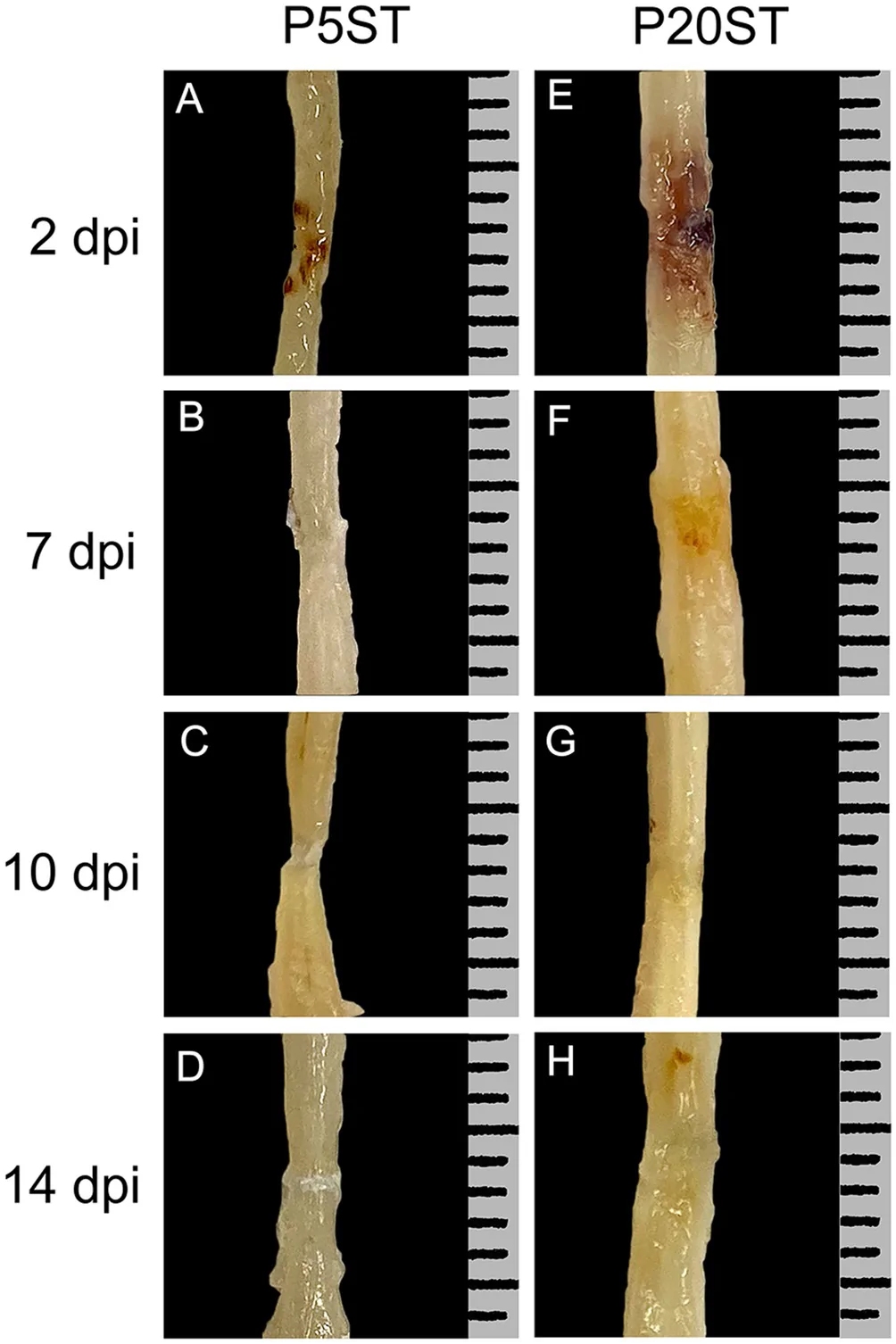
Changes in macroscopic images following total thoracic spinal cord transection at 2 dpi (A), 7 dpi (B), 10 dpi (C), and 14 dpi (D) in the P5ST group, and at 2 dpi (E), 7 dpi (F), 10 dpi (G), and 14 dpi (H) in the P20ST group.

### 3.2 Dynamics of PDGFRβ‐Positive Structures and Collagen I Expression After CST

In the P5ST group, many DAPI‐positive cells had infiltrated into the gap between the proximal and distal stumps by 2 dpi, but only a few PDGFRβ‐positive structures were observed at 2 and 4 dpi (Figure 2A–D). The gap widened by 7 dpi, typically leading to the development of large cavities in the lesion core by 10 dpi. The number of PDGFRβ‐positive structures in the lesion core further decreased, and these cells were only sparsely observed in the remaining lesion core and meningeal tissue after 7 dpi (Figure 2E–J). Collagen I‐positive structures were also sparsely observed in the lesion core at 2 and 4 dpi (Figure 2A–D). Despite the scarcity of PDGFRβ‐positive structures, large bundles of collagen I‐positive fibers connecting the proximal and distal stumps of the spinal cord were occasionally observed in the lesion core (Figure 2E). The collagen I‐positive fibers in these bundles were arranged in the rostrocaudal direction (Figure 2F). Collagen I‐positive structures as well as PDGFRβ‐positive structures were observed in the intact meningeal tissue. Collagen I in the meninges at the incision level was disrupted at 2 dpi (Figure 2A) but remodeled by 4 dpi (Figure 2C). After 7 dpi, the remodeled meninges containing PDGFRβ‐positive structures were prominently thickened (Figure 2E,G,I). The area‐normalized immunofluorescence intensity of PDGFRβ‐positive structures within the lesion core in the P5ST group was 6.90 ± 1.57 at 2 dpi, 6.15 ± 1.47 at 4 dpi, 2.49 ± 0.58 at 7 dpi, 3.25 ± 0.59 at 10 dpi, and 2.22 ± 0.33 at 14 dpi (Figure 4).

**FIGURE 2:**
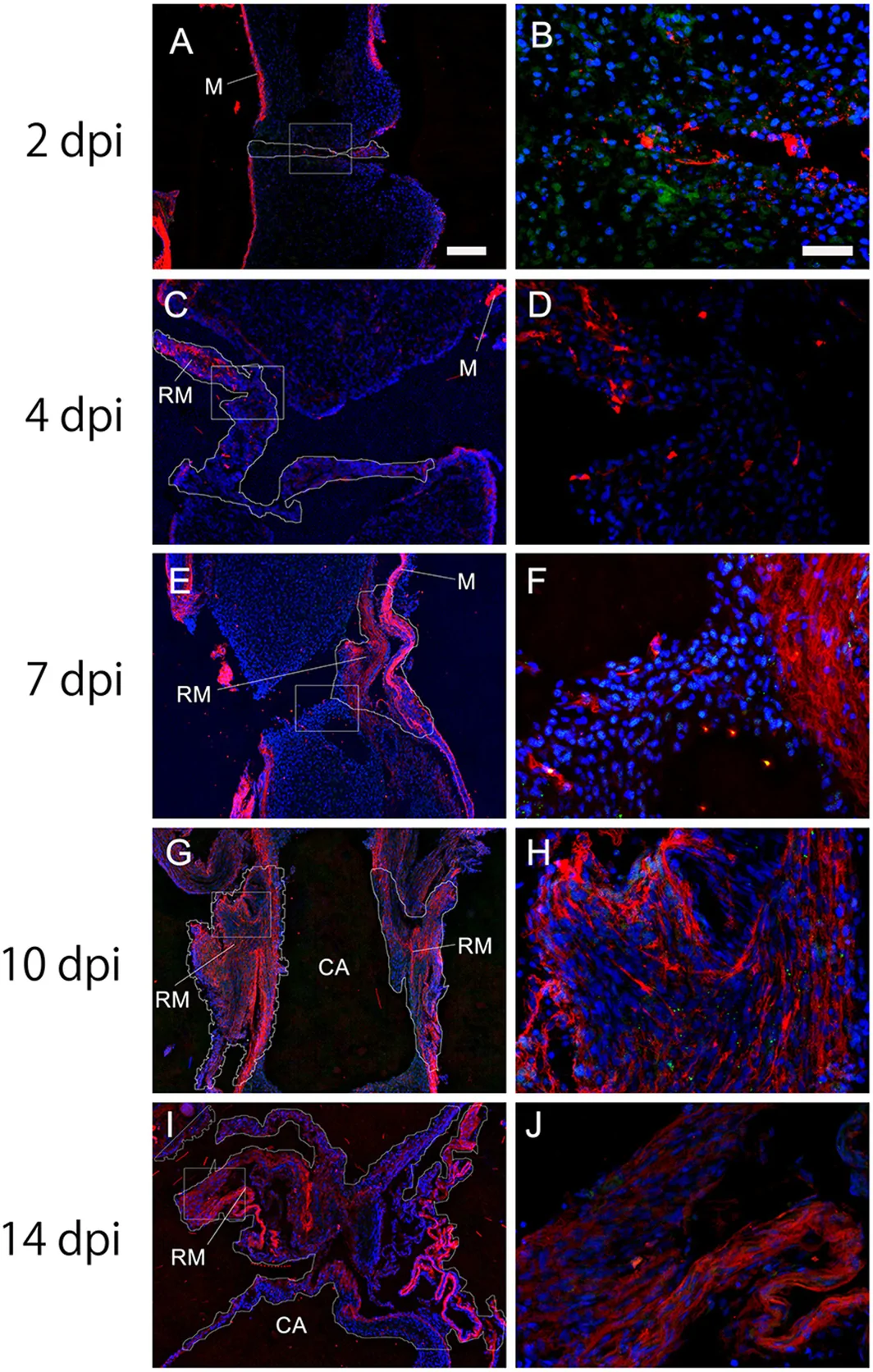
Distribution of PDGFRβ‐positive structures and collagen I in the lesion core following CST in the P5ST group. Triple labeling of PDGFRβ (green), collagen I (red), and DAPI (blue) at 2 dpi (A,B), 4 dpi (C,D), 7 dpi (E,F), 10 dpi (G,H), and 14 dpi (I,J). (A,C,E,G) Overview images of the lesion core. The border of lesion core is indicated by a white line. (B,D,F,H) Enlarged images of the areas marked by rectangles in A, C, E, and G. PDGFRβ‐positive structures were sparse in the lesion core at 2–14 dpi. Collagen I‐positive structures were also sparsely distributed in the lesion core but formed a large bundle connecting the proximal and distal stumps by 7 dpi (arrow in E and F). The gap between the rostral and caudal stumps was larger by 7 dpi, forming large cavities by 10 dpi. The injured meningeal tissue (arrow in A) was remodeled after 4 dpi and prominently thickened after 10 dpi. M, meningeal tissue; CA, cavity; RM, remodeled meninges. Scale bars: 200 μm in A (applies also to C, E, G, and I) and 50 μm in B (applies also to D, F, H, and J).

PDGFRβ‐positive structures were also observed within the lesion core at 2 dpi in the P20ST group (Figure 3A,B). PDGFRβ‐positive structures subsequently increased, spreading throughout the lesion core, peaking at 7 dpi, and then decreasing (Figure 3C–J). Collagen I‐positive structures were also increased after 4 dpi, distributing throughout the lesion core and intermingling with PDGFRβ‐positive structures (Figure 3C–H). At 14 dpi, collagen I‐positive structures remained in the lesion core, despite the decrease of PDGFRβ‐positive structures (Figure 3I,J). The injured meninges of the P20ST group were also remodeled by 4 dpi (Figure 3C). In the P20ST group, unlike the P5ST group, no meningeal thickening was observed (Figure 3E,G). The area‐normalized intensity of PDGFRβ‐positive structures within the lesion core in the P20ST group was 10.71 ± 3.00 at 2 dpi, 21.02 ± 2.75 at 4 dpi, 39.06 ± 4.89 at 7 dpi, 21.67 ± 4.89 at 10 dpi, and 19.44 ± 3.22 at 14 dpi (Figure 4).

**FIGURE 3:**
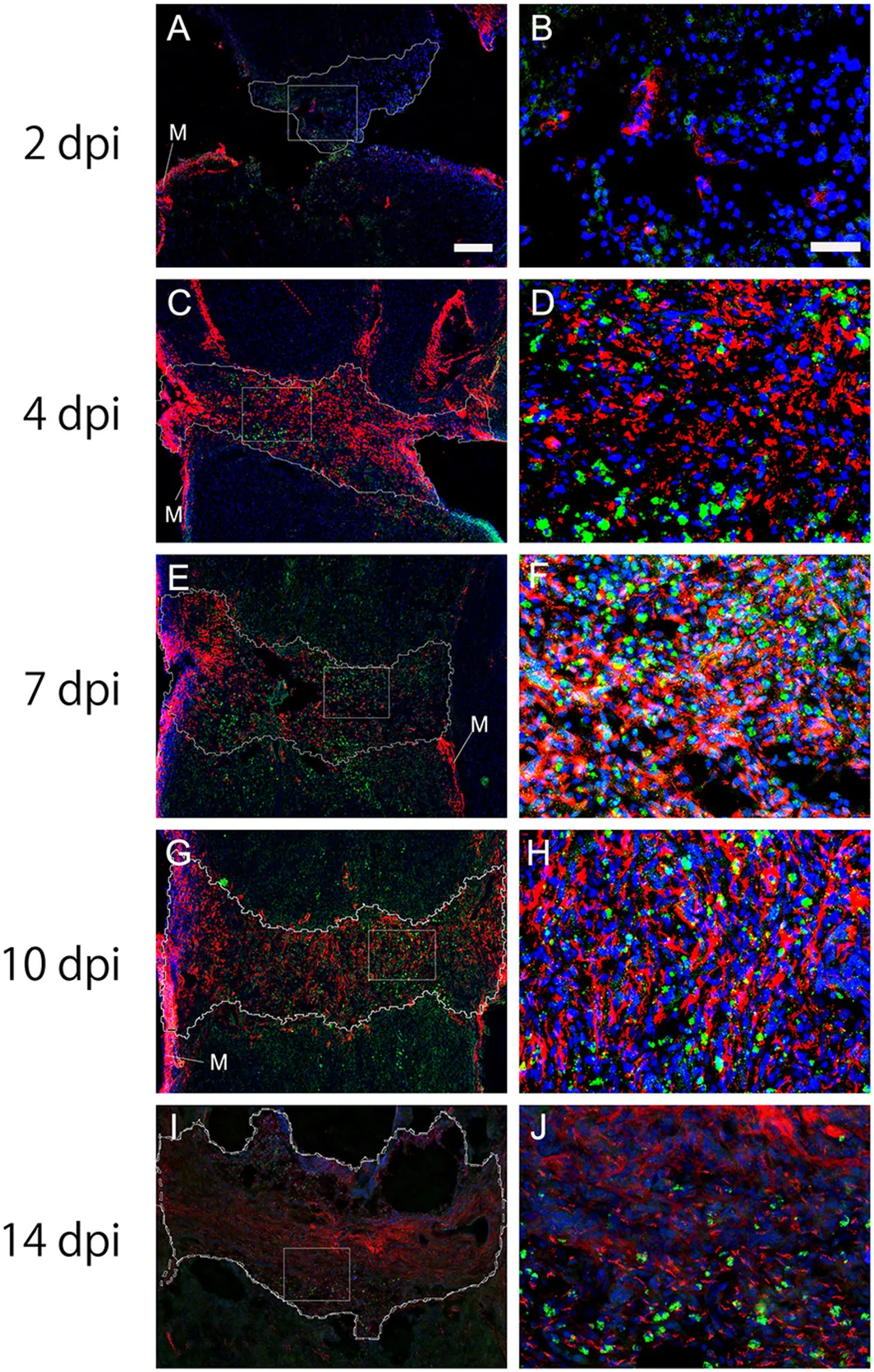
Distribution of PDGFRβ‐positive structures and collagen I in the lesion core following CST in the P20ST group. Triple labeling of PDGFRβ (green), collagen I (red), and DAPI (blue) at 2 dpi (A,B), 4 dpi (C,D), 7 dpi (E,F), 10 dpi (G,H), and 14 dpi (I,J). (A,C,E,G,I) Overview images of the lesion core. The border of lesion core is indicated by a white line. (B,D,F,H,J) Enlarged images of the areas marked by rectangles in A, C, E, G, and I. PDGFRβ‐positive structures and collagen I‐positive structures increased between 4 and 10 dpi in the lesion core, forming fibrous scar tissue. At 14 dpi, collagen I‐positive structures were still observed in the lesion core, whereas PDGFRβ‐positive structures decreased. M, meningeal tissue. Scale bars: 200 μm in A (applies also to C, E, G, and I), and 50 μm in B (applies also to D, F, H, and J).

**FIGURE 4:**
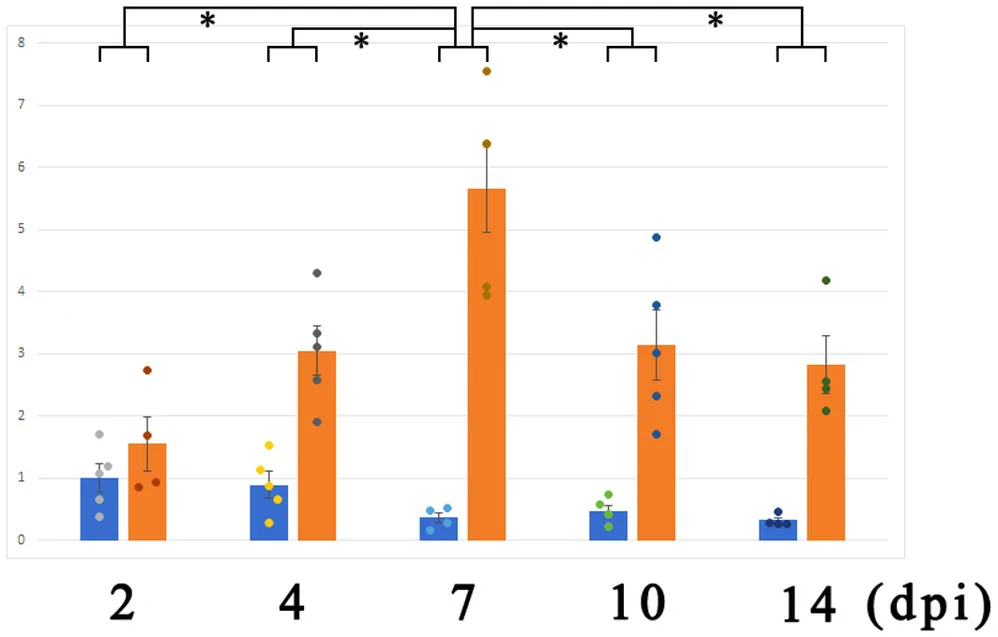
Temporal change in the relative immunofluorescence intensity of PDGFRβ‐positive structures in the lesion core. Blue columns: P5ST group. Orange columns: Values are mean ± SEM The values are relative to the value of the P5ST group at 2 dpi. Significant differences are indicated by *p < 0.05.

Statistical analysis using two‐way ANOVA revealed a significant difference (*F*(1,36) = 107.058, *p* < 0.001) between the P5ST and P20ST groups, and a significant difference (*F*(4,36) = 5.228, *p* = 0.002) in the time after CST. Post hoc analysis using Tukey's HSD revealed significant differences in intensity between 2 and 7 dpi (*p* < 0.001), 4 and 7 dpi (*p* = 0.014), 7 and 10 dpi (*p* = 0.005), and 7 and 14 dpi (*p* = 0.002). An interaction (*F*(4,36) = 8.834, *p* < 0.001) was detected between the CST groups and time after CST. Student's *t* tests revealed significant differences between the P5ST and P20ST groups at 4 dpi (*p* = 0.0014), 7 dpi (*p* = 0.0016), 10 dpi (*p* = 0.0081), and 14 dpi (*p* = 0.0123).

### 3.3 CD11c‐Positive Cell Dynamics After CST

In the P5ST group, CD11c‐positive cells had abundantly infiltrated the lesion core between the rostral and caudal stumps at 2 dpi (Figure 5A), then rapidly decreased to 4 dpi (Figure 5B–E). At 7 dpi and thereafter, large cavities were formed, and few CD11c‐positive cells were observed in the lesion core (Figure 5C–E). They were observed only sparsely in the meningeal tissue (Figure 5D). The cell density at the lesion core was 3.64 ± 1.53 at 2 dpi, 0.57 ± 0.29 at 4 dpi, 0.24 ± 0.04 at 7 dpi, 0.23 ± 0.10 at 10 dpi, and 1.23 ± 0.42 at 14 dpi in the P5ST group (Figure 6).

**FIGURE 5:**
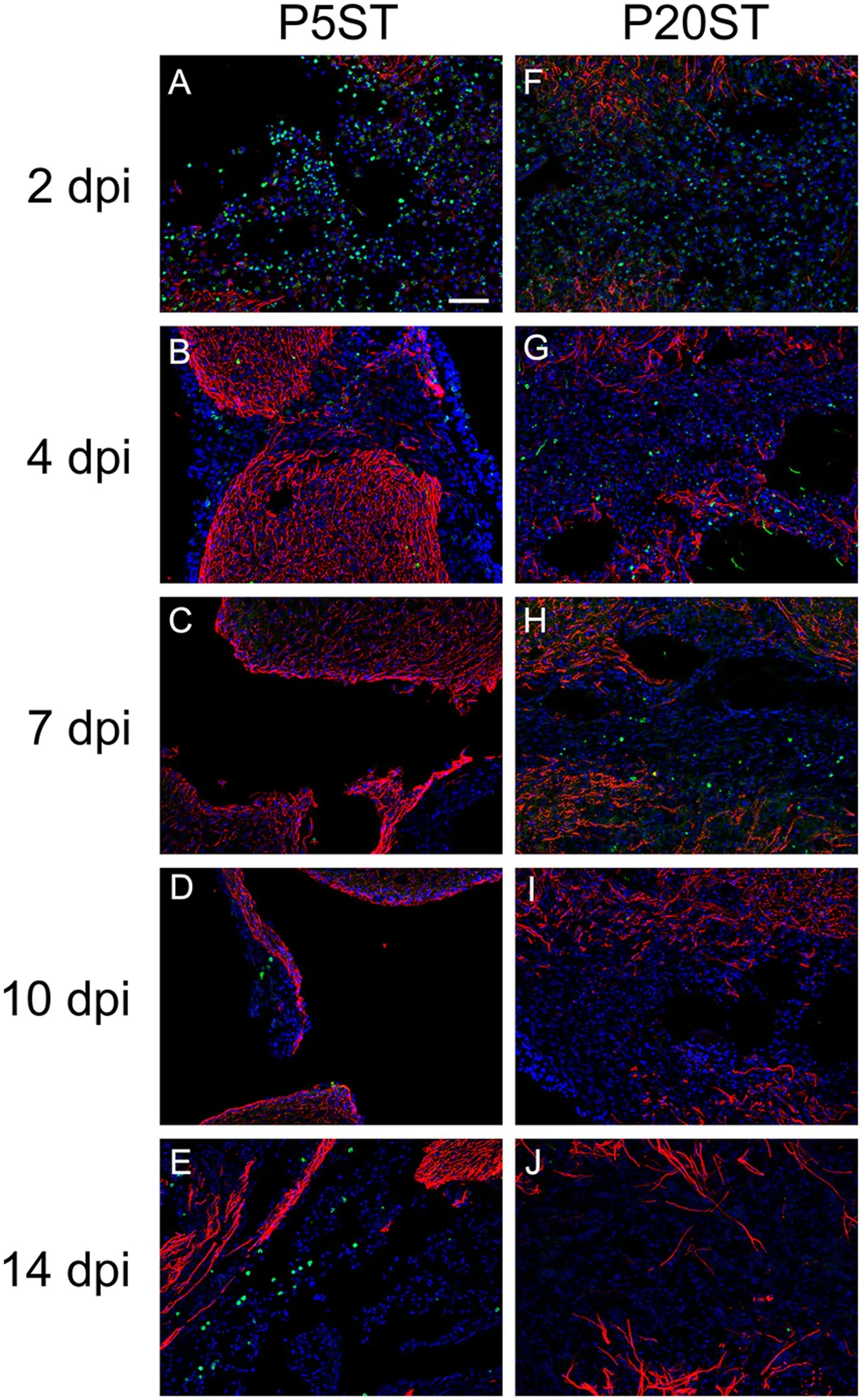
Distribution of CD11c‐positive cells in the lesion core following CST. Triple labeling of CD11c (green), GFAP (red), and DAPI (blue) at 2 dpi (A), 4 dpi (B), 7 dpi (C), 10 dpi (D), and 14 dpi (E) in the P5ST group, and at 2 dpi (F), 4 dpi (G), 7 dpi (H), 10 dpi (I), and 14 dpi (J) in the P20ST group. In both the P5ST and P20ST groups, CD11c‐positive cells in the lesion core were abundant at 2 dpi, then decreased thereafter. Scale bar: 100 μm in A (applies also to B–J).

**FIGURE 6:**
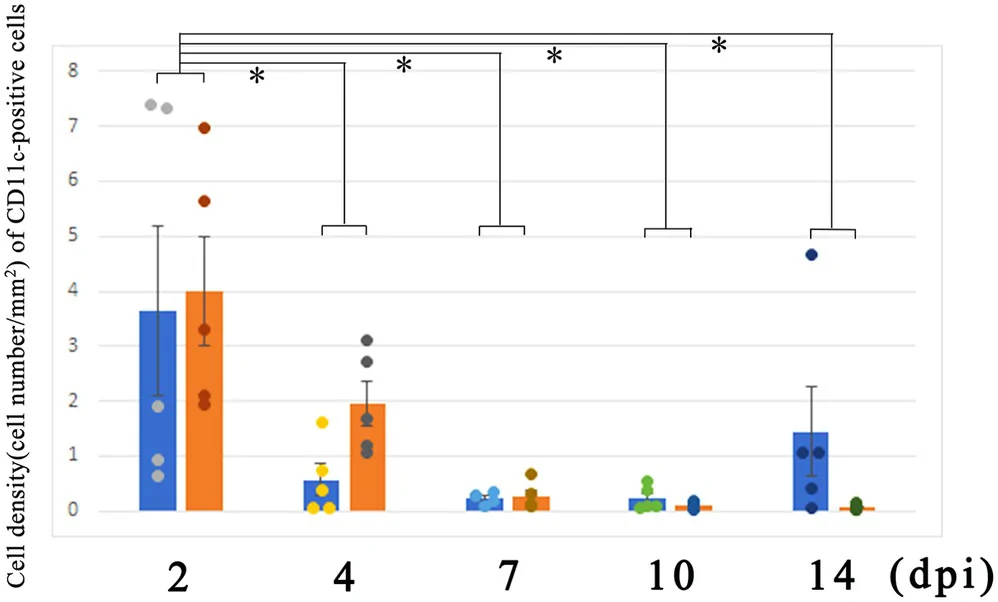
Temporal change in the cell density of CD11c‐positive cells in the lesion core. Blue columns: P5ST group. Orange columns: Values are mean and SEM. Significant differences are indicated by *p < 0.05.

In the P20ST group, CD11c‐positive cells were also abundantly distributed in the lesion core at 2 dpi (Figure 5F). The number of CD11c‐positive cells in the lesion core gradually decreased thereafter, and few were observed at 10 and 14 dpi (Figure 5G–J). The cell density at the lesion core was 4.00 ± 1.00 at 2 dpi, 1.95 ± 0.41 at 4 dpi, 0.26 ± 0.11 at 7 dpi, 0.11 ± 0.03 at 10 dpi, and 0.06 ± 0.02 at 14 dpi in the P20ST group (Figure 6).

Statistical analysis of cell density using two‐way ANOVA revealed no significant difference (*F*(1,40) = 0.015, *p* = 0.902) between the P5ST and P20ST groups, and a significant difference (*F*(4,40) = 10.445, *p* < 0.001) in the time after CST. Post hoc analysis using Tukey's HSD revealed significant differences between 2 and 4 dpi (*p* = 0.003), 2 and 7 dpi (*p* < 0.001), 2 and 10 dpi (*p* < 0.001), and 2 and 14 dpi (*p* < 0.001). There was no interaction (*F*(4,40) = 1.154, *p* = 0.345) between the CST groups and time after CST.

### 3.4 CD163‐Positive Cell Dynamics After CST

Many CD163‐positive cells were scattered in the lesion between the rostral and caudal stumps at 2 dpi in the P5ST group (Figure 7A), and their number further increased by 4 dpi (Figure 7B). Although numerous CD163‐positive cells were still observed in the remaining lesion core at 7 and 10 dpi (Figure 7C,D), the number decreased by 14 dpi (Figure 7E). Many CD163‐positive cells were also observed in the remodeled meningeal tissue (Figure 7B,C,E). The cell density within the lesion core in the P5ST group was 1.29 ± 0.66 at 2 dpi, 3.04 ± 0.32 at 4 dpi, 2.74 ± 0.87 at 7 dpi, 2.84 ± 0.76 at 10 dpi, and 1.14 ± 0.24 at 14 dpi (Figure 8).

**FIGURE 7:**
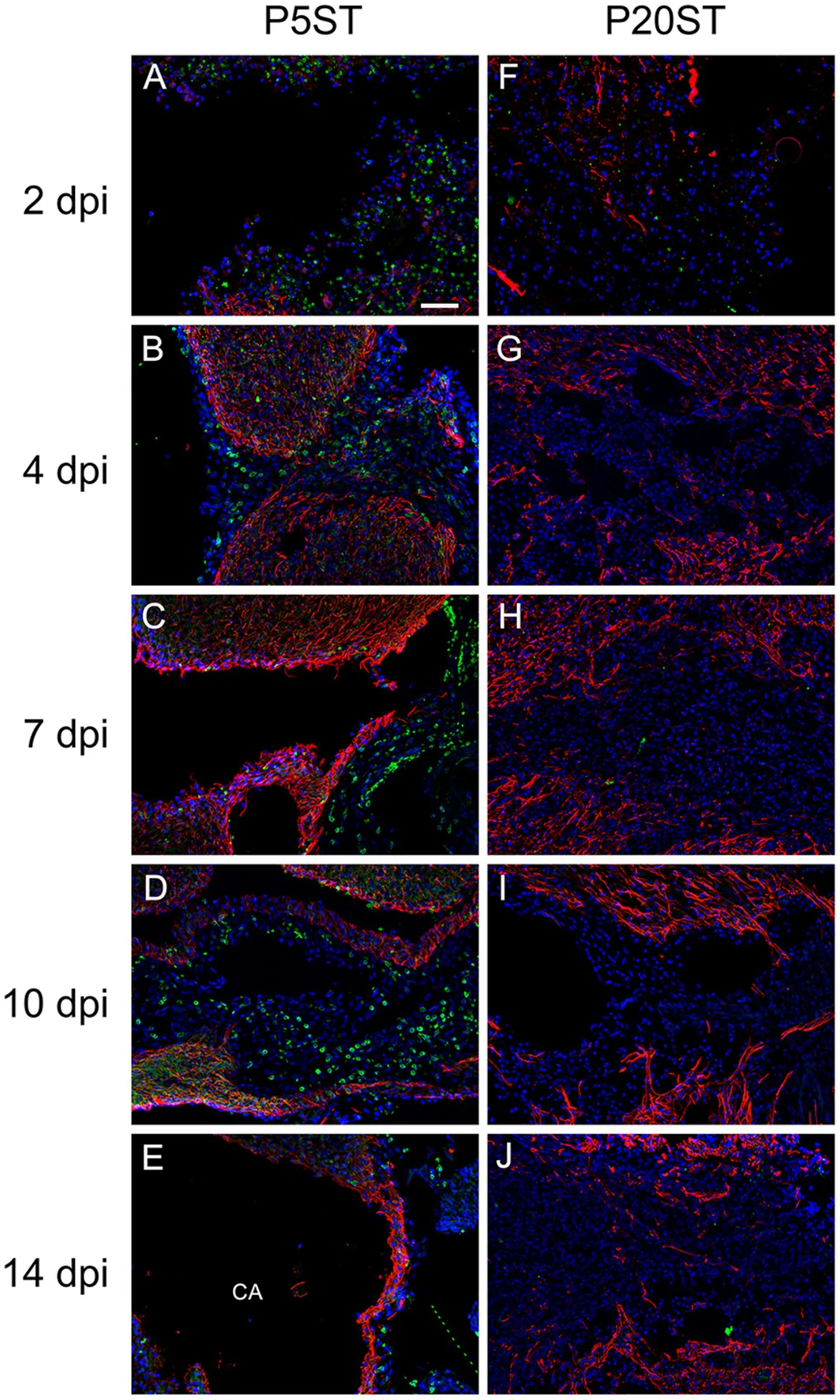
Distribution of CD163‐positive cells in the lesion core following CST. Triple labeling of CD163 (green), GFAP (red), and DAPI (blue) at 2 dpi (A), 4 dpi (B), 7 dpi (C), 10 dpi (D), and 14 dpi (E) in the P5ST group, and at 2 dpi (F), 4 dpi (G), 7 dpi (H), 10 dpi (I), and 14 dpi (J) in the P20ST group. CD163‐positive cells in the P5ST group were abundant in the lesion core at 2 dpi, and further increased between 4 and 10 dpi. In contrast, CD163‐positive cells in the P20 ST group were sparse at 2 dpi and did not increase thereafter. CA, cavity. Scale bar: 100 μm in A (applies also to B–J).

**FIGURE 8:**
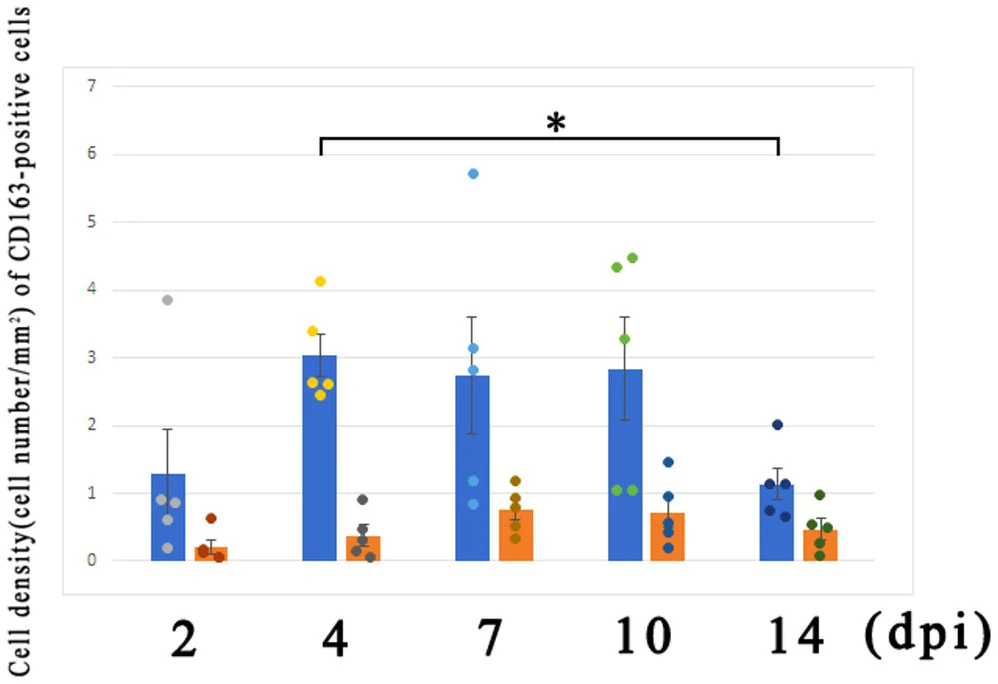
Temporal change in the cell density of CD163‐positive cells in the lesion core. Blue columns: P5ST group. Orange columns: P20ST group. Values are mean ± SEM. Significant differences are indicated by *p < 0.05.

In the P20ST group, CD163‐positive cells were sparsely scattered throughout the lesion core at 2 dpi (Figure 7F); however, unlike in the P5ST group, their number did not increase thereafter (Figure 7H–J). The cell density within the lesion core in the P20ST group was 0.21 ± 0.11 at 2 dpi, 0.38 ± 0.15 at 4 dpi, 0.76 ± 0.15 at 7 dpi, 0.71 ± 0.23 at 10 dpi, and 0.47 ± 0.15 at 14 dpi (Figure 8).

Statistical analysis of cell density using two‐way ANOVA revealed a significant difference (*F*(1,40) = 35.409, *p* < 0.001) between the P5ST and P20ST groups, and a significant difference (*F*(4,40) = 2.754, *p* = 0.041) in time after CST. Post hoc analysis using Tukey's HSD, however, revealed no significant difference in the time after CST. No interaction (*F*(4,40) = 1.604, *p* = 0.192) was detected between the CST groups and time after CST. Subsequent analysis of cell density using one‐way ANOVA in the P5ST group revealed a significant difference (*F*(4) = 5.439, *p* = 0.015). Post hoc analysis using Games–Howell revealed a significant difference between 4 and 14 dpi (*p* = 0.011).

### 3.5 Confirmation of Microglia

Sall1‐positive cells and HEXB‐positive cells were observed in the remodeled meninges of the P5ST group at 2, 4, and 7 dpi. These cells were sparse; however, their density appeared to be lower than that of CD163‐positive cells at the same time points (Figure S1).

## 4 Discussion

The fibrotic scar that forms at the lesion core after adult SCI comprises connective tissue elements such as fibroblasts and ECM and serves to fill the defective tissue, although it may impede regenerating axonal elongation (Klapka and Müller 2006). PDGFRβ is essential for mesenchymal cells to differentiate into fibroblasts and acquire the ability to produce ECM (Gao et al. 2005). The cellular origin of PDGFRβ‐positive mesenchymal cells in the lesion core remains incompletely understood. In mice, PDGFRβ‐positive mesenchymal cells arise from two perivascular cell populations, pericytes, and perivascular fibroblasts, which migrate to the injury site following SCI and contribute to fibrotic scar formation as stromal myofibroblasts (Göritz et al. 2011; Soderblom et al. 2013; Dias et al. 2018, 2021; Holl et al. 2024). In contrast, a recent study in mice demonstrated that PDGFRβ‐positive meningeal fibroblasts contribute more extensively to fibrotic scar formation after CST than perivascular fibroblasts (Xue et al. 2024).

CST in neonatal rats does not result in the formation of a distinct fibrotic scar but rather causes thinning of the lesion core, with the rostral and caudal stumps of the spinal cord connected by a membranous structure (Yuan et al. 2013). Although partial recovery of the somatic and autonomic motor activities occurs spontaneously after the neonatal CST, the appearance of the thinned spinal cord remains unchanged for a long period. In this study, we revealed that the intensity of PDGFRβ‐positive immunofluorescent structures in the lesion core was very low at 2 dpi and subsequently decreased further, suggesting that neonatal CST does not promote fibroblast recruitment or proliferation. In contrast, PDGFRβ‐positive immunofluorescent structures were already frequently observed at 2 dpi in the P20ST group and increased thereafter, suggesting that juvenile CST promotes fibroblast recruitment and proliferation, leading to fibrotic scar formation at the lesion core, similar to that observed after adult CST.

Histologic observation of the lesion core in the P5ST group revealed the presence of PDGFRβ‐positive structures in the remodeled meninges, where ECM such as collagen I was also observed. Large cavities were observed at the lesion core in the P5ST group, but not in the P20ST group. Therefore, fibroblasts present following CST in the P5ST group may contribute to remodeling of connective tissues, such as the meninges, which connect the proximal and distal stumps but may not proliferate within the lesion core to form fibrotic scar tissue. The remodeled meninges gradually thickened, and collagen bundles formed on their inner surface.

CD11c is a marker of M1 macrophages, which infiltrate into the parenchyma of the central nervous system in several pathologic conditions (Griffith et al. 2018; Fujisawa 2020). M1 macrophages that accumulate following SCI secrete several pro‐inflammatory cytokines, including interleukin (IL)‐1β and tumor necrosis factor‐α, to facilitate the removal of damaged tissues (Gensel and Zhang 2015). The present study revealed that CD11c‐positive macrophages accumulated at the lesion core early after CST in both the P5ST and P20ST groups, rapidly diminishing by 4 dpi in the P5ST group and 7 dpi in the P20ST group. These findings indicate that M1 macrophages do not persist for a long time at the injury site, and that the inflammatory phase driven by pro‐inflammatory M1 macrophages also concludes within a short period. Thus, the M1 macrophage dynamics in this study resemble those following injury to somatic organs that undergo complete healing. These findings do not support the conventional view that M1 macrophages persist at the lesion core long after SCI, inhibiting transitions through phases of wound healing and inducing a chronic inflammatory state (Kigerl et al. 2009; Shechter and Schwartz 2013; Gensel and Zhang 2015; Mesquida‐Veny et al. 2021). A rapid decline in M1 macrophages may be a characteristic of CST in both neonatal and juvenile rats. In addition to macrophages, however, CD11c is expressed on dendritic cells, which play a crucial role in the inflammatory response after SCI (Helft et al. 2015; Han et al. 2024). Therefore, the observed decline in CD11c‐positive cells cannot be attributed specifically to changes in M1 macrophages and may also reflect a decrease in dendritic cells at the injury site. Further studies using more specific markers than CD11c may be needed to clarify the dynamics of M1 macrophages after CST.

CD163 is a scavenger surface receptor expressed on M2 macrophages (Abraham and Drummond 2006; Komohara et al. 2006; David and Kroner 2011; Zhang et al. 2012; Franco and Fernández‐Suárez 2015; Zhang et al. 2022). CD163‐positive macrophages are not present in the intact central nervous system parenchyma, but infiltrate and accumulate within the parenchyma under various pathologic conditions including traumatic brain injury (Zhang et al. 2012). CD163 is expressed primarily on M2c macrophages and is considered a representative marker of this subtype (Gensel and Zhang 2015; Lurier et al. 2017; Olmes et al. 2016; Bayat et al. 2023). The localization and roles of CD163‐positive macrophages and M2c macrophages after SCI remain poorly understood. M2c macrophages are primarily associated with the remodeling phase of normal wound healing in somatic organs, whereas their presence after adult SCI remains unclear because normal remodeling process does not properly occur (Gensel and Zhang 2015). The present study revealed that only a small number of CD163‐positive cells were present in the P20ST group, suggesting that, as in adult SCI, M2c macrophages play only a minimal role in tissue healing after juvenile SCI.

The present results in the P5ST group, on the other hand, revealed that numerous CD163‐positive macrophages accumulated at the lesion core by 2 dpi and persisted after 10 dpi. These CD163‐positive macrophage dynamics after SCI are consistent with previously reported M2c macrophage dynamics during normal wound healing, i.e., M2c macrophages are activated early after injury and reach peak activation during the remodeling phase, which begins at approximately 7 dpi (Gensel and Zhang 2015; Lurier et al. 2017; Bayat et al. 2023). These M2c macrophage dynamics may contribute to lesion morphology in the P5ST group. M2c macrophages are induced by stimulation with IL‐10 or transforming growth factor (TGF)‐β and primarily mediate anti‐inflammatory and tissue repair functions (Martinez et al. 2009; Carta et al. 2021). They secrete high levels of matrix metalloproteinase 7 (MMP7), MMP8, and tissue inhibitor of metalloproteinase 1, and express genes associated with angiogenesis, matrix remodeling, and phagocytosis (Lurier et al. 2017; Bayat et al. 2023). M2c macrophages also suppress fibrogenesis and fibrosis in injured tissue (Anders and Ryu 2011; Tang et al. 2017; Luo et al. 2023). Thus, CD163‐positive macrophages in the lesion core following neonatal CST may contribute to suppressing fibroblast recruitment and proliferation, thereby limiting fibrotic scar formation.

CD163 is also expressed in perivascular and meningeal macrophages in the central nervous system (Kim et al. 2006). This is consistent with observation of many CD163‐positive cells in the remodeled meninges of the P5ST group. It is unclear whether these cells possess the characteristics of M2c macrophages, but the present findings suggest that CD163‐positive macrophages are involved in repairing injured tissue, particularly meningeal tissue. CD163‐positive macrophages could contribute to the formation of thickened meningeal tissue and collagen bundles connecting the rostral and caudal stumps by promoting connective tissue remodeling in the residual lesion tissue.

Although microglia were not distinguished from macrophages in the present analysis, their presence in the lesion core was confirmed using microglia‐specific markers. Sall1‐ and HEXB‐positive cells were sparsely distributed within the remodeled meninges, suggesting a more limited accumulation of microglia than CD163‐positive cells. Because fibronectin expression was not evaluated in the present study, it could not be determined whether microglia contribute to fibronectin‐mediated bridge formation, as reported after contusion injury (Li et al. 2020). Nevertheless, the remodeled meninges observed after CST consisted primarily of fibroblasts and collagen, indicating a tissue‐repair response distinct from that reported in neonatal‐contusion injury (Li et al. 2020).

Differences in macrophage dynamics in the lesion core between the P5ST and P20ST groups may contribute to their distinct patterns of tissue repair. In the P5ST group, the prolonged presence of CD163‐positive macrophages was associated with meningeal remodeling and collagen formation, whereas these cells were less prominent in the P20ST group. Conversely, the P20ST group exhibited fibroblast recruitment and proliferation, followed by fibrotic scar formation, suggesting the involvement of macrophage population with different functional properties. Macrophages involved in wound healing promote processes like matrix synthesis, fibrogenesis, and myofibroblast differentiation, ultimately leading to fibrosis in the injured tissue (White and Gomer 2015; Duan et al. 2018; Sapudom et al. 2021; Huang et al. 2024). Although the phenotypes and functions of these macrophage populations were not directly examined in the present study, difference in macrophage responses between the P5ST and P20ST groups may contribute to their distinct patterns of fibrotic scar formation, meningeal remodeling, and cavity formation following CST.

## Author Contributions

**Masahito Takiguchi:** methodology, resources, investigation, validation, formal analysis, visualization. **Reina Tamaji:** conceptualization, investigation, validation, formal analysis, visualization. **Kengo Funakoshi:** data curation, writing – original draft, funding acquisition.

## Funding

This work was supported in part by the Research Clerkship Funding and the Fundamental Research Funding of Yokohama City University.

## Ethics Statement

The protocols of this study were approved by the Institutional Animal Care and Use Committee of the Animal Research Center, Yokohama City University Graduate School of Medicine (approval NO. F‐A‐24‐047).

## Conflicts of Interest

The authors declare no conflicts of interest.

## Supporting information


**Figure S1:** Immunohistochemistry for Sall1 and HEXB, specific microglia markers, at the injury site of P5ST rats at 2 (A,C) and 7 dpi (B,D). (A,B) Double labeling of Sall1 (green) and DAPI (blue). (C,D) Double labeling of HEXB (green) and DAPI (blue). Sall1‐and HEXB‐positive cells were sparsely distributed in the remodeled meninges. Scale bar: 100 μm in D (applies also to A–C).

## Data Availability

Data available on request from the corresponding authors.
